# Bio-Inspired Spotted Hyena Optimizer with Deep Convolutional Neural Network-Based Automated Food Image Classification

**DOI:** 10.3390/biomimetics8060493

**Published:** 2023-10-18

**Authors:** Hany Mahgoub, Ghadah Aldehim, Nabil Sharaf Almalki, Imène Issaoui, Ahmed Mahmud, Amani A. Alneil

**Affiliations:** 1 Department of Computer Science, College of Science & Art at Mahayil, King Khalid University, Muhayil 61321, Saudi Arabia; 2 Department of Information Systems, College of Computer and Information Sciences, Princess Nourah Bint Abdulrahman University, P.O. Box 84428, Riyadh 11671, Saudi Arabia; 3 Department of Special Education, College of Education, King Saud University, Riyadh 12372, Saudi Arabia; 4 Unit of Scientific Research, Applied College, Qassim University, Buraydah 51425, Saudi Arabia; 5 Research Center, Future University in Egypt, New Cairo 11835, Egypt; 6 Department of Computer and Self Development, Preparatory Year Deanship, Prince Sattam bin Abdulaziz University, Al-Kharj 11942, Saudi Arabia

**Keywords:** computer vision, deep convolutional neural network, machine learning, food image classification, spotted hyena optimizer

## Abstract

Food image classification, an interesting subdomain of Computer Vision (CV) technology, focuses on the automatic classification of food items represented through images. This technology has gained immense attention in recent years thanks to its widespread applications spanning dietary monitoring and nutrition studies to restaurant recommendation systems. By leveraging the developments in Deep-Learning (DL) techniques, especially the Convolutional Neural Network (CNN), food image classification has been developed as an effective process for interacting with and understanding the nuances of the culinary world. The deep CNN-based automated food image classification method is a technology that utilizes DL approaches, particularly CNNs, for the automatic categorization and classification of the images of distinct kinds of foods. The current research article develops a Bio-Inspired Spotted Hyena Optimizer with a Deep Convolutional Neural Network-based Automated Food Image Classification (SHODCNN-FIC) approach. The main objective of the SHODCNN-FIC method is to recognize and classify food images into distinct types. The presented SHODCNN-FIC technique exploits the DL model with a hyperparameter tuning approach for the classification of food images. To accomplish this objective, the SHODCNN-FIC method exploits the DCNN-based Xception model to derive the feature vectors. Furthermore, the SHODCNN-FIC technique uses the SHO algorithm for optimal hyperparameter selection of the Xception model. The SHODCNN-FIC technique uses the Extreme Learning Machine (ELM) model for the detection and classification of food images. A detailed set of experiments was conducted to demonstrate the better food image classification performance of the proposed SHODCNN-FIC technique. The wide range of simulation outcomes confirmed the superior performance of the SHODCNN-FIC method over other DL models.

## 1. Introduction

Food image detection and identification are the existing research subjects in the domain of Computer Vision (CV). “Food” is one of the developing areas of interest for the CV community as well as multimedia [1], whereas image detection and identification remain a highly significant problem in the medical field as well. In the literature, a new food recording tool called “FoodLog” has been developed that supports users to record their daily meals with the aid of an image recovery technique [2]. However, it is extremely challenging to perform food image analyses. For instance, the identification of food products in images is still a challenging process due to low inter-class variance and high intra-class variance [3]. Furthermore, many food classes have not yet been effectively classified. Therefore, automated food detection is a developing area of research not only in the image recognition domain but also in social media research [4]. A significant number of researchers paid attention to this domain due to its improving advantages from a medical viewpoint [5]. Automated food identification tools can support and facilitate decision-making methods in terms of calorie calculation, food quality detection, diet monitoring systems to overcome obesity, etc. [6]. In general, food is naturally distorted and has a broad difference in appearance [7]. Food images may have high intra-class and low inter-class variances, owing to which standard techniques may not be able to detect complex features in the images. This drawback in the food identification process makes it a challenging task since complex features cannot be detected using conventional methods [8].

Recently, several developments have occurred in the domain of dietary valuation depending on multimedia approaches, e.g., food image analysis [9]. In the literature, an automated image-based nutritional assessment technique was proposed in which the technique had the following key stages: food image identification, recognition of food products, weight or quantity valuation, and lastly, nutritional and caloric value assessment [10]. In recent years, developments in Machine Learning (ML), image processing, and specifically Convolutional Neural Networks (CNN), and Deep-Learning (DL) techniques have heavily benefited the image classification and detection processes, comprising the issue of food image identification [11]. Researchers have developed diverse phases of food detection systems, despite which it remains challenging to find a satisfactory and efficient solution for food identification and classification with high accuracy. This is because there exist extensive types of food products and extremely complicated hybrid food products in food images [12]. Therefore, it is tremendously challenging to detect all food items accurately since a variety of food items can appear similar in terms of shape, color, or context, and are not even differentiable to the human eye [13].

Given this background, the current research article develops the Bio-Inspired Spotted Hyena Optimizer with a Deep Convolutional Neural Network-based Automated Food Image Classification (SHODCNN-FIC) approach. The presented SHODCNN-FIC method exploits the DL model with hyperparameter tuning approaches for the classification of food images. To achieve this, the SHODCNN-FIC method exploits the DCNN-based Xception model to derive the feature vectors. In addition to this, the SHODCNN-FIC technique uses the SHO algorithm for the optimal hyperparameter selection of the Xception model. The SHODCNN-FIC technique uses the Extreme Learning Machine (ELM) model for the detection and classification of food images. A detailed set of experiments was conducted to illustrate the better food image classification performance of the SHODCNN-FIC technique. The key contributions of the current study are summarized below.

(a) The development of an automated SHODCNN-FIC algorithm, including Xception feature extraction, SHO-based parameter tuning, and ELM-based classification for food classification. To the best of the authors’ knowledge, the SHODCNN-FIC approach has never been reported in the literature.
(b) The development of a new technique, i.e., SHODCNN-FIC, by combining bio-inspired optimization and DL for automatic food image classification. The proposed technique is highly useful in many real-time applications involving dietary analysis and restaurant menu management.
(c) The SHODCNN-FIC leverages the power of deep learning using the DCNN-based Xception model for extracting the feature vectors from food images. Furthermore, the optimum fine-tuning of the hyperparameters for the Xception model, using the SHO technique, improves the performance of the DL model by fine-tuning its parameters.
(d) The application of the ELM model for the actual detection and classification of food images. ELM is known for its high accuracy and fast training features in different machine-learning tasks.

The rest of the paper is organized as follows. Section 2 discusses the related works, and Section 3 details the proposed model. Then, Section 4 provides the analytical results, and Section 5 concludes the paper.

## 2. Related Works

Shah and Bhavsar [14] introduced the depth-restricted-CNN (DRCNN) method in which the Transfer Learning (TL) technique was applied to a few frameworks, such as the Alexnet, Resnet-50, Inceptionv3, VGG16, and the VGG19 framework. This method was presented as a Batch Normalization (BN) approach that heavily enhances performance with a lower number of parameters. Chopra and Purwar [15] introduced a food image detection system composed of CNN, GA, and PSO to improve outcomes. CNN, as an approach, was utilized in this study for the classification of food images. The reason for supplementing the CNN technique with GA and PSO is to ensure an efficient classification outcome. In the literature [16], an enhanced VGG16 framework was proposed through a food classification technique. This approach employed the Asymmetric Convolution Block (ACB) to change the convolution kernels and enhance the effectiveness of the standard technique. This technique also involved BN and pooling layers to enrich the normalization. The attention mechanism should be integrated with the CNN technique due to its complications, such as higher texture similarity, complex context, and contextual intervention.

Chopra and Purwar [17] developed the Squirrel Search Algorithm (SSA) to provide optimum solutions for multiple thresholds. This technique implemented the CNN method to identify food images. Then, the study suggested that the Enhanced SSA (ESSA) increases food detection accuracy. Yadav and Chand [18] recommended automatic food classification techniques with the help of the DL algorithm. In this study, both VGG-16 and SqueezeNet CNNs were exploited for the classification of food images. These networks demonstrated significantly high effectiveness due to two tasks, namely fine-tuning the hyperparameters and data augmentation. The developed VGG16 framework then enhanced the performance of the automated food image classification process. In the study conducted earlier [19], the CNN approach was introduced and employed to recognize and classify food images. A pre-trained Inceptionv3 CNN algorithm was implemented using TL to stimulate the original customized CNN model. By utilizing the pre-trained method, the learning approach increased, and therefore, more proficient results were achieved. Therefore, data augmentation must be executed on the training set, since it enhances the performance.

Pan et al. [20] recommended a novel classification technique based on the DL approach for the automatic identification of food items. A combinational CNN (CBNet) was created with a subnet integrating method in this study. Primarily, two different NNs were employed to learn important features. Secondarily, a highly developed feature fusion element combined the features from sub-networks. Shermila et al. [21] introduced a new DL-based Food Item Classification (DEEPFIC) method in which the image was processed using the sigmoid stretching algorithm to improve the quality of the images and eliminate the noise. Afterward, the preprocessed image was segmented by employing the Improved Watershed Segmentation (IWS2) technique. In this study, the RNN approach was utilized for the extraction of the features, which were then normalized through the dragonfly algorithm. The Bi-LSTM was employed in this study for the classification of food items.

Though existing automatic food image classification algorithms are valuable, these methods have critical shortcomings that need to be resolved. One important limitation is that these methods often have a narrow scope in identifying food items from certain cultural contexts or cuisines, therefore resulting in poor generalization whenever it encounters unconventional or diverse dishes. Furthermore, these models struggle when handling variations in food presentation techniques, including changes in angles, lighting, or plating styles, which are common in real-time scenarios. Therefore, a research need exists to develop a highly effective and efficient hyperparameter optimization method, particularly a customized one for food image classification tasks. This is because the hyperparameter tuning process is a crucial aspect in enhancing model performance. This involves the exploration of novel techniques or the adaptation of existing ones to overcome the unique challenges posed by food image datasets. The hyperparameter tuning process ultimately affects the generalization ability and performance of the models. In this scenario, the DL model is extremely complicated and has various hyperparameters, namely batch sizes, learning rates, regularization strengths, and layer depths, among others. This hyperparameter considerably affects the performance of the model in terms of learning from data and its capability to fit patterns while preventing over-fitting issues. Without accurate tuning, the DL model converges slowly, becomes trapped in a sub-optimal solution, or fails to adapt to certain features of the dataset. By systematically adjusting the hyperparameters through techniques such as random search, grid search, or metaheuristic optimization algorithms, DL algorithms can be fine-tuned to accomplish high accuracy, fast convergence rate, and best generalization. These outcomes make the model highly effective in different applications. Addressing these research gaps can advance the field of automatic food image classification using hyperparameter tuning and contribute to the development of highly efficient, accurate, and interpretable models with real-time applications in fields such as food waste reduction, dietary analysis, and restaurant menu management.

## 3. The Proposed Model

**Figure 1:**
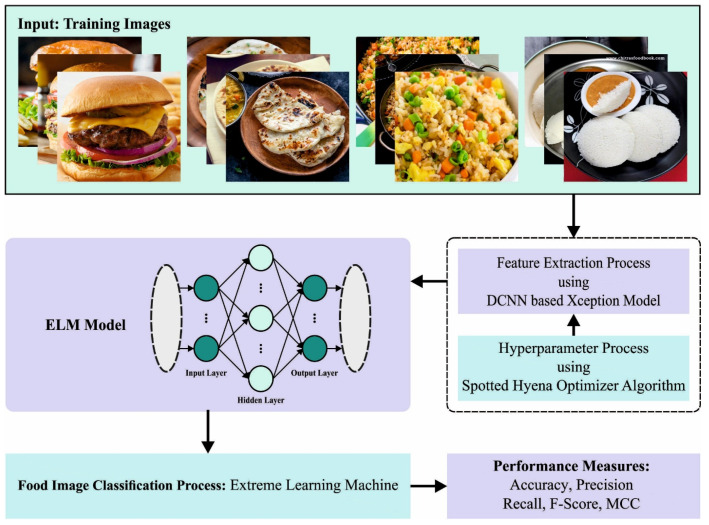
Overall flow of the SHODCNN-FIC algorithm.

The current research article is focused on the design and development of an automated food image detection and classification algorithm named the SHODCNN-FIC approach. The main objective of the SHODCNN-FIC method is to recognize and classify food images into distinct types. The presented SHODCNN-FIC technique exploits the DL model with hyperparameter tuning strategies for the classification of food images. It involves different stages of operations, namely Xception, SHO-based hyperparameter tuning, and ELM classification. Figure 1 shows the entire procedure of the SHODCNN-FIC algorithm.

### 3.1. Feature Extraction Using Xception Model

The SHODCNN-FIC technique uses the DCNN-based Xception model to derive the feature vectors. The CNN model has proved to be an extraordinary implementer of different image-grouping problems in various fields [22]. The concept of sharing the load in CNN makes the image segmentation process a difficult one by finding the high-level components in the images and diminishing the dissipating tendency problem. The development of the CNN technique incorporates the related layer, convolution layer, and pooling layer. The convolution layer deals with channels, whereas the chief aim is to eliminate the features from the images. Both pooling and convolution layers yield low performances when looking at and holding the basic data in food images. The final layer is the related layer that uses ReLU and takes a certain level component from the food image to gather them into different classifications with marks.

In the XceptionNet model, the conventional convolutional layers are exchanged for depth-wise separable convolution layers. The CNN feature map enables cross-channel and decoupling spatial correlation, whereas the mapping of both correlations is added to the basic operations of the network. Finally, the XceptionNet replaces the main structure of the Inception model. XceptionNet, with 36 convolution layers, is divided into 14 modules. First, the actual image is transformed into defining the possibility contained over different input channels to arrive at the unified images. The following scheme exploits 11 depth-wise convolution layers. 

### 3.2. Hyperparameter Tuning Using the SHO Algorithm

The SHO algorithm is applied for the hyperparameter tuning process. This technique is based on the hunting strategy of the hyena predation model [23]. It comprises four phases: searching, siege, hunting, and attacking. It continuously approaches and encircles the prey after recognizing its location. The individual search for a target is the optimal searching point, and the rest update their positions [24]. The distance equation between the prey and the spotted hyenas is given in Equation (1).

(1) $$D_{h}=\left|B\cdot P_{P}\left(t\right)-P\left(t\right)\right|$$

where  B shows the coefficient vector, $D_{h}$ signifies the distance between the captured prey and the searched individual, and t denotes the iteration count. $P_{P}$ and P show the position of the target and the individuals searched for during t iteration.

(2) $$P\left(t+1\right)=P_{P}\left(t\right)-E\cdot D_{h}$$

where E signifies the coefficient vector. The individual search location at the t+1 iteration is related to the target point and the distance between them. Equations (1) and (2) contain a coefficient vector and the expression is as follows:

(3) $$\left\{\begin{array}{l} B=2\cdot r_{d1}\\ E=2h\cdot r_{d2} \end{array}\right.$$

where $r_{d1}$ and $r_{d2}$ are random numbers that lie in the range [0, 1]; h indicates the control factor that drops linearly from 5 to 0 as follows:

(4) $$\left|h\right|=5-\left[I_{inter}\left(\frac{5}{M_{inter}}\right)\right]\;$$

where $M_{inter}$ denotes the maximal iteration count; $I_{inter}$ shows the natural numbers except 0. The spotted hyenas frequently engage in groups to encircle the target. Assume that a better-searched individual is much closer to the target, whereas the rest defines the location of the better-searched individuals as the target location, which forms a cluster and cooperatively moves toward the optimum point location. The computation equation for this scenario is attained from the succeeding formula:

(5) $$\left\{\begin{array}{l} D_{h}=\left|B\cdot P_{P}-P_{k}\right|\\ P_{k}=P_{h}-E\cdot D_{h} \end{array}\right.$$

where $P_{h}$ indicates the optimum location for the hyena group; $P_{k}$ shows the location of the residual hyenas.

(6) $$\left\{\begin{array}{c} C_{h}=P_{k}+P_{k+1}+\ldots +P_{k+N}\\ N=C_{nos}\left(P_{h},P_{h+1},P_{h+2},\ldots ,P_{h}+M\right) \end{array}\right.$$

where $\;C_{h}$ shows a set of N optimum solutions; N refers to the number of spotted hyenas; and $C_{nos}$ indicates the number of solutions attained. The coefficient vector E changes continuously, whereas the control factor h gradually decreases. Once the absolute value of E becomes less than 1, then it is the attack moment. Otherwise, it continues to search for prey. The computation equation for this scenario is as follows:

(7) $$P\left(t+1\right)=\frac{C_{h}}{N}$$

**Figure 2:**
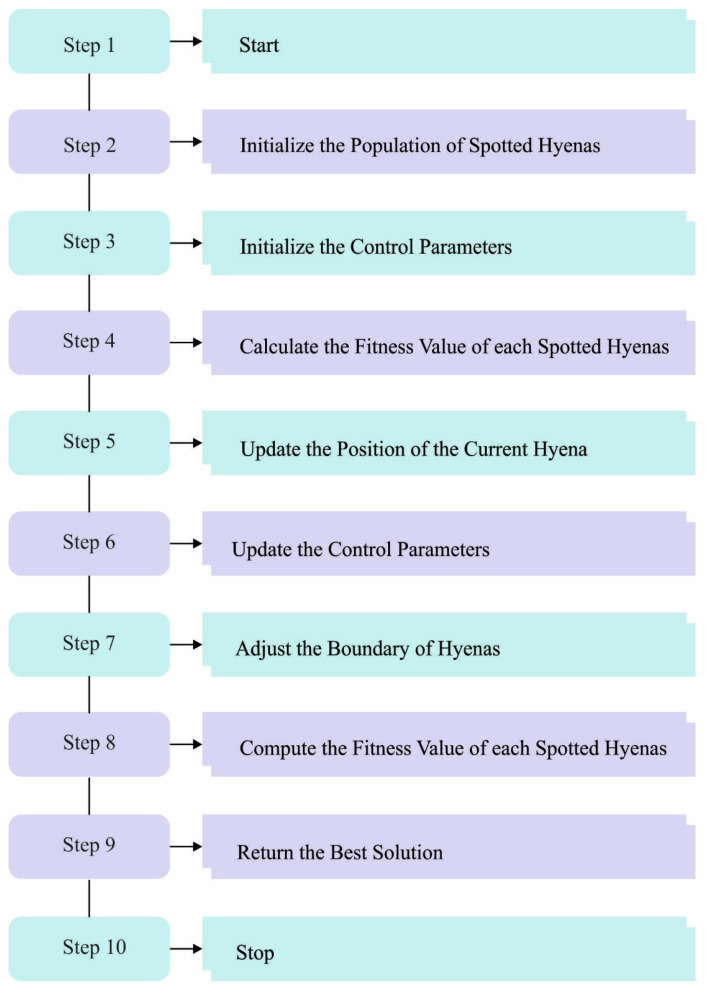
Steps involved in the SHO algorithm.

$C_{h}$ is the optimum search individual set, where the individuals disperse and pursue the target. The condition is that $\left|E\right|$ is higher than 1, after which the distance between the target and spotted hyenas is forcefully limited. Extending the search phase might assist in finding a better hunting position and ensure the successful implementation of global search. Figure 2 depicts the steps involved in the SHO algorithm.

The SHO system progresses an FF to offer the highest classification solution. It expresses a positive integer to exemplify the optimal solution of the candidate performance. The reduction in classifier errors is assumed to be FF.

(8) $$fitness\left(x_{i}\right)=ClassifierErrorRate\left(x_{i}\right)=\frac{No.\;of\;misclassified\;instances\;}{Total\;no.\;of\;instances\;}\times 100$$

### 3.3. Image Classification Using the ELM Model

In this study, the ELM algorithm has been applied to the food image classification process. ELM is an FFNN model for ML that provides various benefits compared to other techniques, including RBFNN and BPNN [25]. It does not need adjustment of the structural parameters, which makes it an easier and highly effective one. In ELM, the weights connected between the hidden and input layers, along with the threshold of the HL neurons, are randomly generated and do not require adjustment during training. Consider that there exist N training instances $\left(X_{i},\;Y_{i}\right),$ where $1\leq i\leq N,\;X_{j}={\left[X_{i1},\;X_{i2},\;\cdots ,\;X_{in}\right]}^{T}\in R^{n}$ refers to the input vector of the *i*th sample, and $Y_{i}={\left[y_{i1},y_{i2},\;\cdots ,y_{im}\right]}^{T}\in R^{m}$ indicates the output vector of the *i*th samples as follows

(9) $$\Sigma_{l}^{f}\beta_{f}g\left(w_{f}X_{i}+b_{f}\right)=T_{i}$$

In Equation (9), $g\left(x\right)$ denotes the activation function;$\;T_{i}={\left[t_{i1},\;t_{i2},\;\cdots ,\;t_{im}\right]}^{T}$ implies the output vector of the *i*th sample. $w_{f}={\left[w_{f1},w_{f2},\cdots ,w_{fn}\right]}^{T}$ indicates the input weight; $b_{f}$ shows the threshold of f−th HL neuron; and $\beta_{f}=\left[\beta_{f1},\beta_{f2},\cdots ,\beta_{fm}\right]$ represents the output weight. The objective of the ELM technique is to minimize the output error as follows:

(10) $$\Sigma_{i=1}^{N}\left|\left|T_{i}-Y_{i}\right|\right|=0\;$$

(11) Hβ=Y

where $H=\left[\begin{array}{lll} g\left(W_{1}X_{1}+b_{1}\right) & \ldots & g\left(W_{l}X_{1}+b_{l}\right)\\ \;\vdots & \ddots & \vdots \\ g\left(W_{1}X_{N}+b_{1}\right) & \ldots & g\left(W_{l}X_{N}+b_{l}\right) \end{array}\right]$ signifies the layer output matrix of the network; $\beta ={\left[\beta_{1}^{T},\beta_{2}^{T},\;\cdots ,\beta_{l}^{T}\right]}_{l\times m}^{T}$; and $Y={\left[Y_{1}^{T},Y_{2}^{T},\;\cdots ,\;T_{N}^{T}\right]}_{N\times m}^{T}.$ The output weight β is attained by resolving the least-square solution as follows:

(12) $$\min\;=\left|\left|H\beta -Y\right|\right|$$

(13) $$\hat{\beta }=H^{+}Y\;$$

Here, the generalized inverse matrix of the output matrix H is represented as $\;H^{+}$.

## 4. Results and Discussion

**Figure 3:**
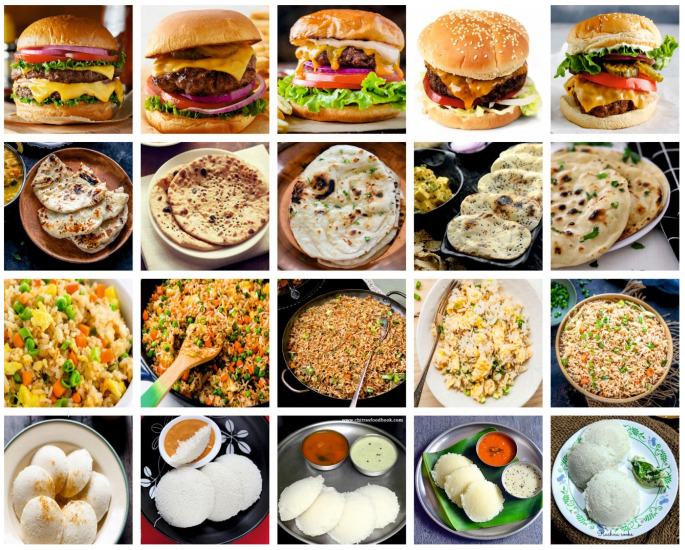
Sample images.

The proposed model was simulated using the Python 3.8.5 release. The proposed model was executed on a PC configured with specifications as follows: i5-8600k, GeForce 1050Ti 4 GB, 16 GB RAM, 250 GB SSD, and 1 TB HDD. The food classification outcomes of the SHODCNN-FIC algorithm were tested using the Indian food classification dataset [26]. The dataset includes a total of 1800 samples under six classes, as defined in Table 1. Figure 3 represents some of the sample images.

**Figure 4:**
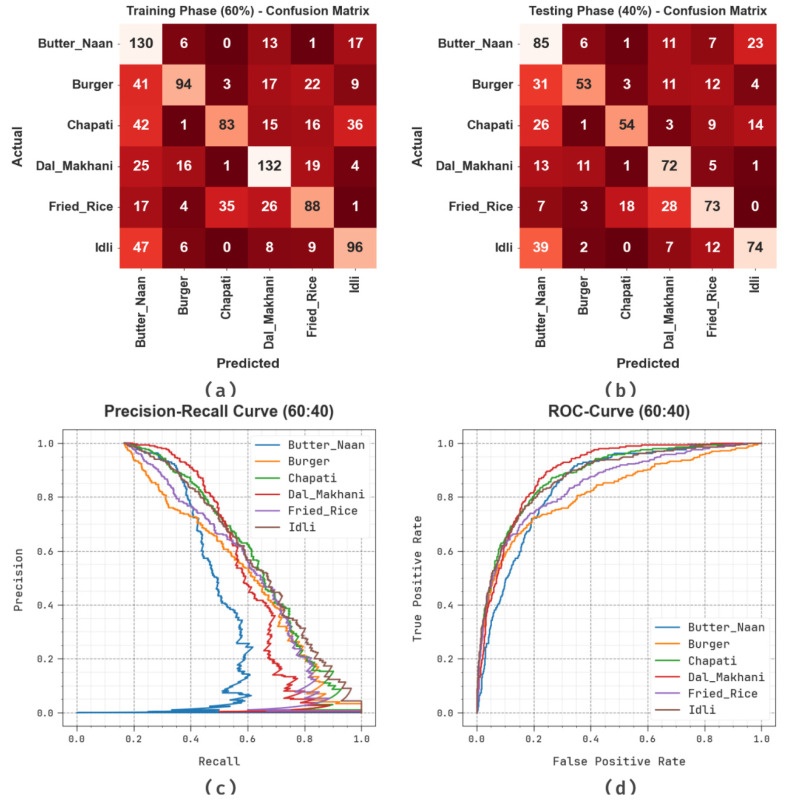
60:40 of TR set/TS set (**a**,**b**) Confusion matrices, (**c**) PR_curve, and (**d**) ROC.

Figure 4 illustrates the classification outcomes of the SHODCNN-FIC method on 60:40 of the TR set/TS set. Figure 4a,b depict the confusion matrix generated by the SHODCNN-FIC approach. The outcome indicates that the SHODCNN-FIC method detected and categorized all six class labels. Likewise, Figure 4c demonstrates the PR examination results of the SHODCNN-FIC method. The figure infers that the SHODCNN-FIC technique attained the maximum PR outcome under all six classes. Lastly, Figure 4d shows the ROC examination outcomes of the SHODCNN-FIC system. The figure shows that the SHODCNN-FIC technique achieved promising outcomes with maximum ROC values under all six class labels.

**Figure 5:**
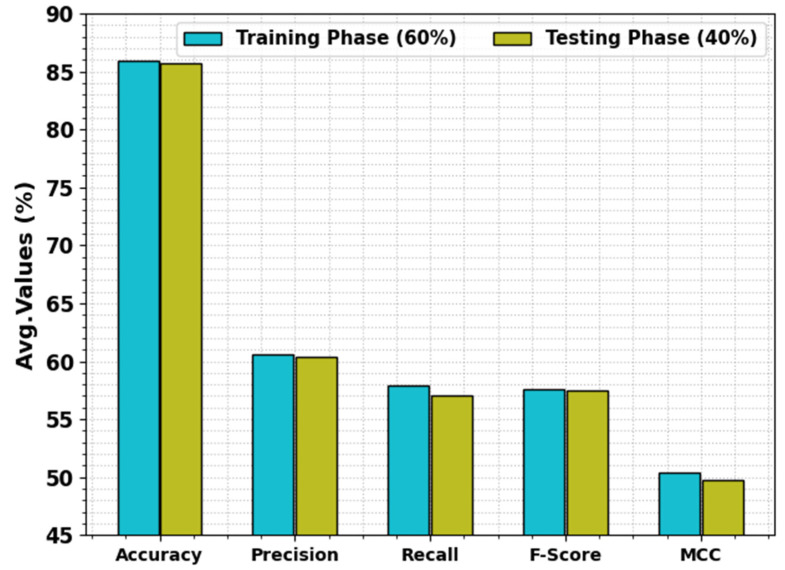
Average values of the SHODCNN-FIC algorithm at 60:40 TR set/TS set.

The food classification results of the SHODCNN-FIC technique with 60:40 of TR set/TS set are reported in Table 2 and Figure 5. The outcomes infer the proficient performance of the SHODCNN-FIC technique on different food classes. On the 60% TR set, the SHODCNN-FIC technique attained the average $accu_{y}$, $prec_{n}$, $reca_{l}$, $F_{score}$, and MCC values of 85.90%, 60.55%, 57.95%, 57.54%, and 50.42%, respectively. Also, on the 40% TS set, the SHODCNN-FIC method accomplished the average $accu_{y}$, $prec_{n}$, $reca_{l}$, $F_{score}$, and MCC values of 85.69%, 60.39%, 57.10%, 57.49%, and 49.79%, respectively.

**Figure 6:**
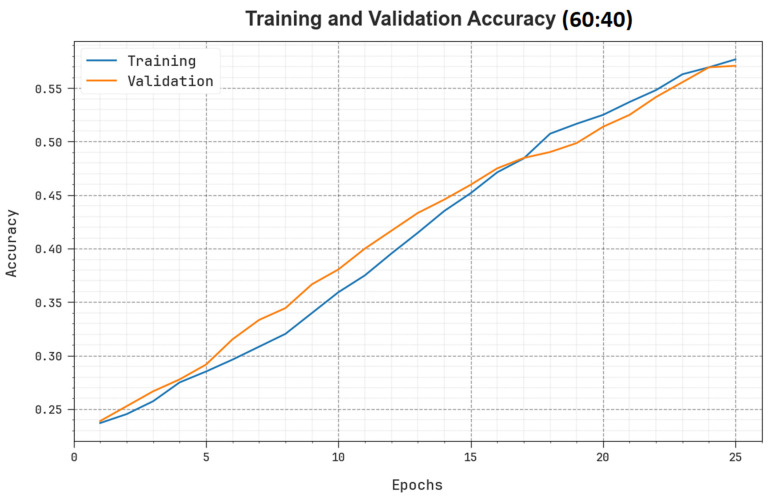
$Accu_{y}$ curve of the SHODCNN-FIC algorithm at 60:40 of the TR set/TS set.

To evaluate the performance of the SHODCNN-FIC method on 60:40 TR set/TS set, the TR and TS $accu_{y}$ curves were plotted and are shown in Figure 6. The TR and TS $accu_{y}$ values illustrate the performance of the SHODCNN-FIC technique over various number of epochs. The figure shows meaningful insights into the learning task and the generalization abilities of the SHODCNN-FIC method. With an increase in the number of epochs, both TR and TS $accu_{y}$ curves improved. The SHODCNN-FIC technique attained improved testing accuracy, which can detect patterns in the TR and TS datasets.

**Figure 7:**
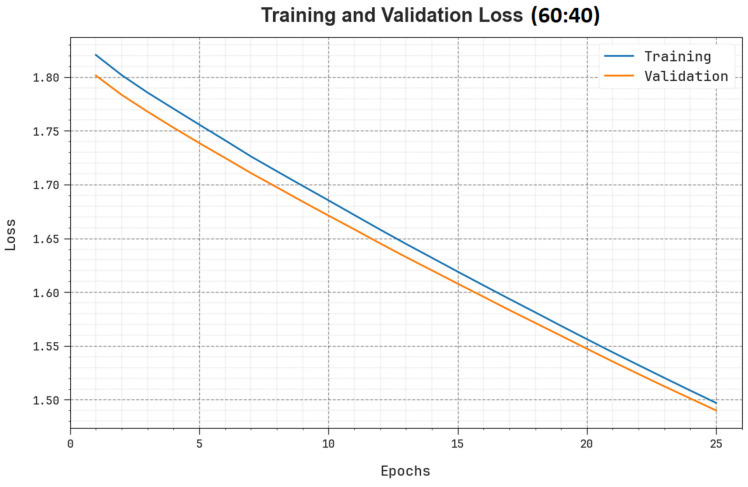
Loss curve of the SHODCNN-FIC algorithm at 60:40 of the TR set/TS set.

Figure 7 displays the overall TR and TS loss values of the SHODCNN-FIC method on 60:40 of the TR set/TS set over a different number of epochs. The TR loss outcomes show that the model’s loss reduced over an increasing number of epochs. Primarily, the loss values were reduced as the model modified the weight to minimize the prediction error on TR and TS datasets. The loss curves illustrate the extent to which the model fits the training data. Both TR and TS loss values steadily decreased, and this shows that the SHODCNN-FIC technique effectually learned the patterns exhibited in the TR and TS datasets. The SHODCNN-FIC approach adjusted the parameters to minimize the discrepancy between the prediction and the original training label.

**Figure 8:**
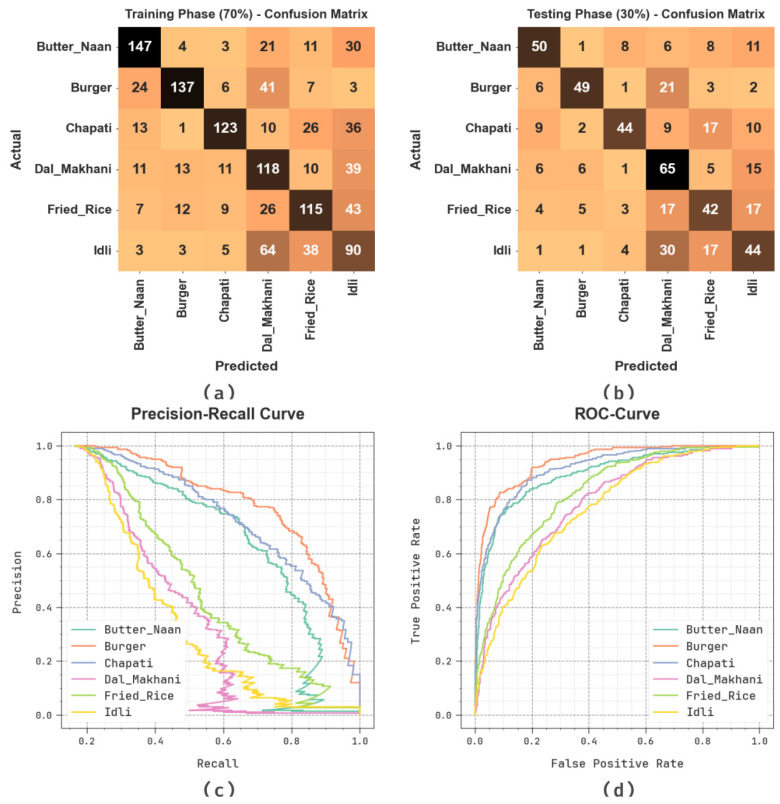
70:30 of TR set/TS set (**a**,**b**) Confusion matrices, (**c**) PR_curve, and (**d**) ROC.

Figure 8 shows the classification outcomes of the SHODCNN-FIC method at 70:30 of the TR set/TS set. Figure 8a,b show the confusion matrix generated by the SHODCNN-FIC technique. The outcome indicates that the SHODCNN-FIC method detected and categorized all six class labels. Likewise, Figure 8c demonstrates the PR examination outcomes of the SHODCNN-FIC method. The figure infers that the SHODCNN-FIC technique attained the maximum PR performance under all six classes. Lastly, Figure 8d depicts the ROC examination outcomes of the SHODCNN-FIC approach. The figure portrays the promising performance of the SHODCNN-FIC approach with maximum ROC values under all six class labels.

**Figure 9:**
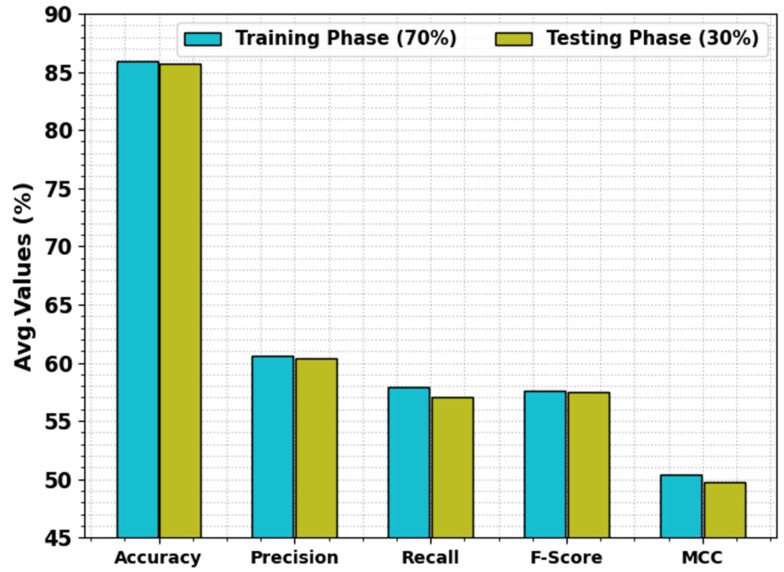
Average values of the SHODCNN-FIC algorithm at 70:30 TR set/TS set.

The food classification results of the SHODCNN-FIC technique with 70:30 of the TR set/TS set are reported in Table 3 and Figure 9. The outcomes found the proficient performance of the SHODCNN-FIC method on different food classes. On the 70% TR set, the SHODCNN-FIC technique achieved average $accu_{y}$, $prec_{n}$, $reca_{l}$, $F_{score}$, and MCC values of 85.98%, 60.95%, 57.79%, 58.68%, and 50.76%, respectively. Also, on the 30% TS set, the SHODCNN-FIC technique yielded average $accu_{y}$, $prec_{n}$, $reca_{l}$, $F_{score}$, and MCC values of 84.81%, 58.08%, 54.51%, 55.32%, and 46.88%, respectively.

**Figure 10:**
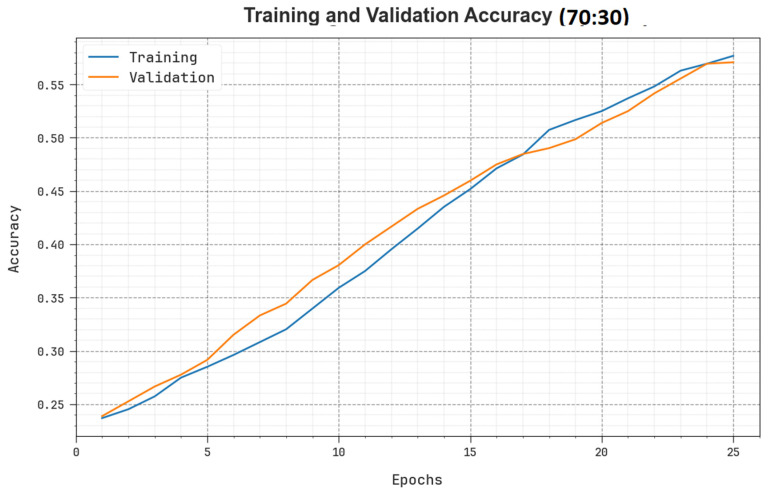
$Accu_{y}$ curve of the SHODCNN-FIC algorithm at 70:30 of the TR set/TS set.

To assess the performance of the SHODCNN-FIC method on the 70:30 TR set/TS set, the TR and TS $accu_{y}$ curves were determined and are shown in Figure 10. The TR and TS $accu_{y}$ curves illustrate the performance of the SHODCNN-FIC technique over several epochs. The figure offers meaningful insights into the learning task and generalization capabilities of the SHODCNN-FIC model. With an increase in the number of epochs, the TR and TS $accu_{y}$ curves were enhanced. It can be observed that the SHODCNN-FIC model obtained enhanced testing accuracy, which can detect the patterns in both TR and TS datasets.

**Figure 11:**
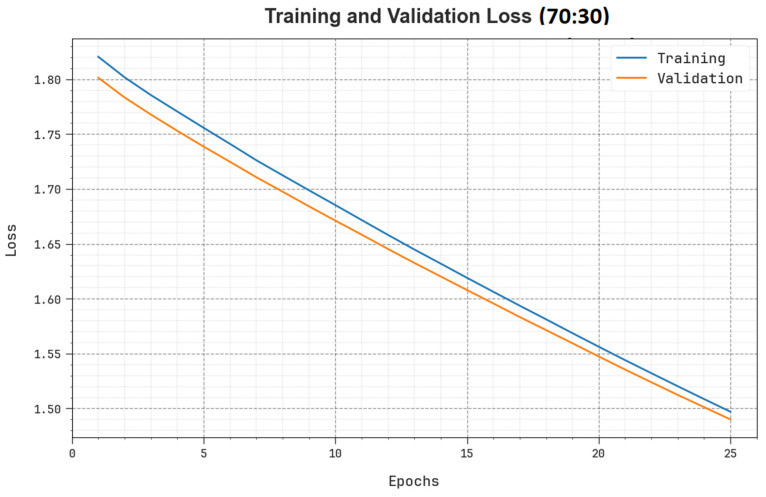
Loss curve of the SHODCNN-FIC algorithm at 70:30 of the TR set/TS set.

Figure 11 shows the overall TR and TS loss values of the SHODCNN-FIC method at 70:30 of the TR set/TS set over a varying number of epochs. The TR loss values illustrate that the model loss reduced over an increasing number of epochs. Primarily, the loss values were reduced as the technique modified the weight to minimize the prediction error on TR and TS data. The loss curves show the extent to which the model fits the training data. Both TR and TS loss values steadily reduced, which shows that the SHODCNN-FIC model effectually learned the patterns displayed in both TR and TS data. The SHODCNN-FIC method adjusted the parameters to minimize the discrepancy between the predicted and the original training label.

**Figure 12:**
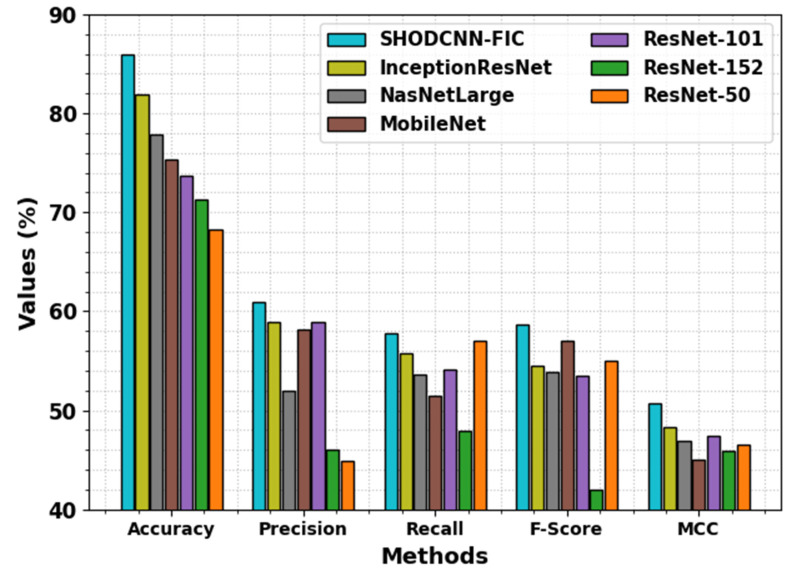
Comparative analysis outcomes of the SHODCNN-FIC algorithm and other recent methods.

In Table 4 and Figure 12, the overall comparative analysis outcomes between the proposed SHODCNN-FIC system and other approaches are given. The outcomes show that the ResNet50 model achieved the worst results, whereas the NASNetLarge, MobileNet, ResNet101, and ResNet152 models obtained slightly closer performances. Meanwhile, the InceptionResNet model gained a considerably high performance. However, the SHODCNN-FIC technique demonstrated promising performance with the maximum $accu_{y}$, $prec_{n}$, $reca_{l}$, $F_{score}$, and MCC values of 85.98%, 60.95%, 57.79%, 58.68%, and 50.76% respectively.

**Figure 13:**
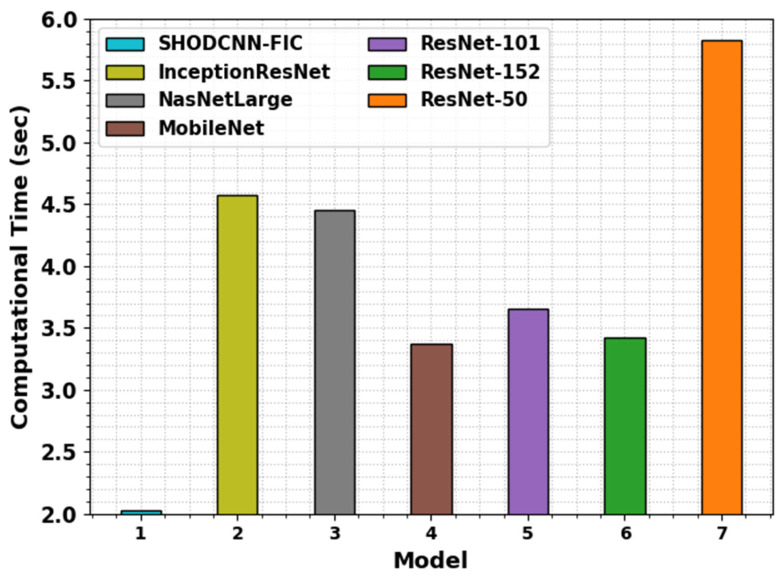
CT outcomes of the SHODCNN-FIC algorithm and other recent methods.

The Computation Time (CT) analysis outcomes of the SHODCNN-FIC technique and other existing DL approaches are demonstrated in Table 5 and Figure 13. The outcomes show the enhanced classification results of the SHODCNN-FIC technique with a minimal CT of 2.03 s. At the same time, it can be observed that the SHODCNN-FIC technique exhibits an enhanced food image classification outcome.

## 5. Conclusions

This paper designs an automated food image detection and classification algorithm named SHODCNN-FIC. The main objective of the SHODCNN-FIC technique is to recognize and classify distinct types of food images. The presented SHODCNN-FIC technique exploits the DL model with hyperparameter tuning strategies for the classification of food images. It involves different stages of operations, namely the Xception, SHO-based hyperparameter tuning, and the ELM classification. To accomplish this, the SHODCNN-FIC technique employs a DCNN-based Xception model to derive feature vectors. In addition, the SHODCNN-FIC technique uses the SHO approach for the selection of the optimum hyperparameters for the Xception model. The SHODCNN-FIC technique uses the ELM model for both the detection and classification of food images. A detailed set of experiments was conducted to demonstrate the enhanced food image classification performance of SHODCNN-FIC. The extensive simulation values portray the improved performance of the SHODCNN-FIC method over other DL approaches. In the future, the SHODCNN-FIC approach could be used to handle multi-modal inputs, such as the integration of image data with textual descriptions or nutritional information. This could enable a highly comprehensive and accurate food recognition and classification system. Future work should focus on adapting SHODCNN-FIC to a real-time basis. Edge computing environments are increasingly relevant, especially for applications like dietary monitoring or mobile food recognition apps.

## Figures and Tables

**Table 1:** Details on the database.

Class	No. of Samples
Butter_Naan	300
Burger	300
Chapati	300
Dal_Makhani	300
Fried_Rice	300
Idli	300
Total Samples	1800

**Table 2:** Food classification outcomes of the SHODCNN-FIC algorithm at 60:40 TR set/TS set.

Class	$Accu_{y}$	$Prec_{n}$	$Reca_{l}$	$F_{score}$	MCC
Training Phase (60%)					
Butter_Naan	80.65	43.05	77.84	55.44	47.53
Burger	88.43	74.02	50.54	60.06	54.91
Chapati	86.20	68.03	43.01	52.70	46.73
Dal_Makhani	86.67	62.56	67.01	64.71	56.55
Fried_Rice	86.11	56.77	51.46	53.99	45.91
Idli	87.31	58.90	57.83	58.36	50.88
Average	85.90	60.55	57.95	57.54	50.42
Testing Phase (40%)					
Butter_Naan	77.22	42.29	63.91	50.90	38.19
Burger	88.33	69.74	46.49	55.79	50.73
Chapati	89.44	70.13	50.47	58.70	53.77
Dal_Makhani	87.36	54.55	69.90	61.28	54.45
Fried_Rice	85.97	61.86	56.59	59.11	50.74
Idli	85.83	63.79	55.22	59.20	50.88
Average	85.69	60.39	57.10	57.49	49.79

**Table 3:** Food classification outcomes of the SHODCNN-FIC algorithm at 70:30 TR set/TS set.

Class	$Accu_{y}$	$Prec_{n}$	$Reca_{l}$	$F_{score}$	MCC
Training Phase (70%)					
Butter_Naan	89.92	71.71	68.06	69.83	63.82
Burger	90.95	80.59	62.84	70.62	66.07
Chapati	90.48	78.34	58.85	67.21	62.64
Dal_Makhani	80.48	42.14	58.42	48.96	38.04
Fried_Rice	85.00	55.56	54.25	54.89	45.90
Idli	79.05	37.34	44.33	40.54	28.09
Average	85.98	60.95	57.79	58.68	50.76
Testing Phase (30%)					
Butter_Naan	88.89	65.79	59.52	62.50	56.09
Burger	91.11	76.56	59.76	67.12	62.71
Chapati	88.15	72.13	48.35	57.89	52.70
Dal_Makhani	78.52	43.92	66.33	52.85	41.09
Fried_Rice	82.22	45.65	47.73	46.67	36.02
Idli	80.00	44.44	45.36	44.90	32.68
Average	84.81	58.08	54.51	55.32	46.88

**Table 4:** Comparative analysis outcomes of the SHODCNN-FIC algorithm and other recent approaches.

Methods	$Accu_{y}$	$Prec_{n}$	$Reca_{l}$	$F_{score}$	MCC
SHODCNN-FIC	85.98	60.95	57.79	58.68	50.76
InceptionResNet	81.91	58.97	55.75	54.54	48.34
NasNetLarge	77.91	51.97	53.67	53.87	46.94
MobileNet	75.36	58.17	51.47	56.97	44.97
ResNet101	73.7	58.97	54.15	53.51	47.38
ResNet152	71.31	45.97	47.97	41.97	45.93
ResNet50	68.27	44.95	56.97	54.97	46.53

**Table 5:** CT outcomes of the SHODCNN-FIC algorithm and other recent methods.

Model	Computational Time (s)
SHODCNN-FIC	2.03
InceptionResNet	4.57
NasNetLarge	4.45
MobileNet	3.37
ResNet-101	3.65
ResNet-152	3.42
ResNet-50	5.83

## Data Availability

Data sharing is not applicable to this article as no dataset was generated during the current study.

## References

[1] VijayaKumari G., Vutkur P., Vishwanath P. (2022). Food classification using transfer learning technique. Glob. Transit. Proc..

[2] Termritthikun C., Jamtsho Y., Muneesawang P., Zhao J., Lee I. (2023). Evolutionary neural architecture search based on efficient CNN models population for image classification. Multimed. Tools Appl..

[3] Chun M., Jeong H., Lee H., Yoo T., Jung H. (2022). Development of Korean Food Image Classification Model Using Public Food Image Dataset and Deep Learning Methods. IEEE Access.

[4] Chen W., Song R. (2003). A new deep learning-based food recognition system for the mobile terminal. Proceedings of the 2023 IEEE 12th Data-Driven Control and Learning Systems Conference (DDCLS).

[5] He L., Cai Z., Ouyang D., Bai H. (2022). Food Recognition Model Based on Deep Learning and Attention Mechanism. Proceedings of the 2022 8th International Conference on Big Data Computing and Communications (BigCom).

[6] Li J., Chen W., Zhu Y., Xuan K., Li H., Zeng N. (2023). Intelligent detection and behavior tracking under ammonia nitrogen stress. Neurocomputing.

[7] Wu P., Wang Z., Li H., Zeng N. (2023). KD-PAR: A knowledge distillation-based pedestrian attribute recognition model with multi-label mixed feature learning network. Expert Syst. Appl..

[8] Liu M., Wang Z., Li H., Wu P., Alsaadi F.E., Zeng N. (2023). AA-WGAN: Attention augmented Wasserstein generative adversarial network with application to fundus retinal vessel segmentation. Comput. Biol. Med..

[9] Mezgec S., Seljak B.K. (2021). Deep neural networks for image-based dietary assessment. JoVE J. Vis. Exp..

[10] Nr D., Gk D.S., Kumar Pareek D.P. A Framework for Food recognition and predicting its Nutritional value through Convolution neural network. Proceedings of the International Conference on Innovative Computing & Communication (ICICC) 2022.

[11] Alahmari S.S., Salem T. (2022). Food State Recognition Using Deep Learning. IEEE Access.

[12] Tasci E. (2020). Voting combinations-based ensemble of fine-tuned convolutional neural networks for food image recognition. Multimedia Tools Appl..

[13] Du J., Coumba B.Y., Jin X. Senegalese Food Recognition for Tourism Information Using Convolution Neural Network. Proceedings of the 2021 5th International Conference on Electronic Information Technology and Computer Engineering.

[14] Shah B., Bhavsar H. (2023). Depth-restricted convolutional neural network—A model for Gujarati food image classification. Vis. Comput..

[15] Chopra M., Purwar A. Food Image Recognition by Optimizing CNN with PSO and GA. Proceedings of the 2022 Fourteenth International Conference on Contemporary Computing 2022.

[16] Liu H., Gong H., Ding X. (2021). Food image recognition algorithm based on improved VGG16. Proceedings of the 2021 IEEE 2nd International Conference on Information Technology, Big Data and Artificial Intelligence (ICIBA).

[17] Chopra M., Purwar A. (2023). Food recognition using enhanced squirrel search optimisation algorithm and convolutional neural network. Int. J. Data Anal. Tech. Strateg..

[18] Yadav S., Chand S. (2021). Automated food image classification using deep learning approach. Proceedings of the 2021 7th International Conference on Advanced Computing and Communication Systems (ICACCS).

[19] Chaitanya A., Shetty J., Chiplunkar P. (2023). Food Image Classification and Data Extraction Using Convolutional Neural Network and Web Crawlers. Procedia Comput. Sci..

[20] Pan L., Li C., Pouyanfar S., Chen R., Zhou Y. (2020). A Novel Combinational Convolutional Neural Network for Automatic Food-Ingredient Classification. Comput. Mater. Contin..

[21] Shermila P.J., Ahilan A., Shunmugathammal M., Marimuthu J. (2023). DEEPFIC: Food item classification with calorie calculation using dragonfly deep learning network. Signal Image Video Process..

[22] Ogundokun R.O., Li A., Babatunde R.S., Umezuruike C., Sadiku P.O., Abdulahi A.T., Babatunde A.N. (2023). Enhancing Skin Cancer Detection and Classification in Dermoscopic Images through Concatenated MobileNetV2 and Xception Models. Bioengineering.

[23] Dhiman G., Kumar V. (2017). Spotted hyena optimizer: A novel bio-inspired based metaheuristic technique for engineering applications. Adv. Eng. Softw..

[24] Yang X., Cheng L. (2023). Hyperspectral Image Pixel Classification based on Golden Sine and Chaotic Spotted Hyena Optimization Algorithm. IEEE Access..

[25] Dong X., Xu H., Cao H., Cui T., Sun Y. (2023). Temperature Compensation of Wind Tunnel Balance Signal Detection System Based on IGWO-ELM. Sensors.

[26] https://www.kaggle.com/datasets/l33tc0d3r/indian-food-classification.

